# Uncovering SNP and indel variations of tetraploid cottons by SLAF-seq

**DOI:** 10.1186/s12864-017-3643-4

**Published:** 2017-03-23

**Authors:** Chao Shen, Xin Jin, De Zhu, Zhongxu Lin

**Affiliations:** National Key Laboratory of Crop Genetic Improvement, College of Plant Science & Technology, Huazhong Agricultural University, Wuhan, 430070 Hubei China

**Keywords:** Tetraploid cotton, SLAF-seq, Single-nucleotide polymorphisms, Insertions/deletions, Phylogeny

## Abstract

**Background:**

Cotton (*Gossypium* spp.), as the world’s most utilized textile fibre source, is an important, economically valuable crop worldwide. Understanding the genomic variation of tetraploid cotton species is important for exploitation of the excellent characteristics of wild cotton and for improving the diversity of cotton in breeding. However, the discovery of DNA polymorphisms in tetraploid cotton genomes has lagged behind other important crops.

**Results:**

A total of 111,795,823 reads, 467,735 specific length amplified fragment (SLAF) tags and 139,176 high-quality DNA polymorphisms were identified using specific length amplified fragment sequencing (SLAF-seq), including 132,880 SNPs and 6,296 InDels between the reference genome (TM-1) and the five tetraploid cotton species. Intriguingly, gene ontology (GO) enrichment analysis revealed that a number of significant terms were related to reproduction in *G. barbadense* acc. 3–79. Based on the new data sets, we reconstructed phylogenetic trees that showed a high concordance to the phylogeny of diploid and polyploid cottons. A large amount of interspecific genetic variations were identified, and some of them were validated by the single-strand conformation polymorphism (SSCP) method, which will be applied in introgression genetics and breeding with *G. hirsutum* cv. Emian22 as the receptor and the other species as donors.

**Conclusions:**

Using SLAF-seq, a large number of DNA polymorphisms were identified. The comprehensive analysis of DNA polymorphisms provided invaluable insights into the different tetraploid cotton species. More importantly, the identification of numerous interspecific genetic variations provides the basis and is very practical for future introgression breeding. The results presented herein provide a valuable genomic resource for new insights into the genetics and breeding of cotton.

**Electronic supplementary material:**

The online version of this article (doi:10.1186/s12864-017-3643-4) contains supplementary material, which is available to authorized users.

## Background

Cotton is the most important natural textile fibre and is a significant oilseed crop, providing approximately 35% of the total fibre used in the world [1], profoundly affecting the world economy and changing human daily life. China is the world’s largest raw cotton producer and consumer, providing two-thirds of the world’s cotton together with India and the United States [1]. The cotton genus consists of over 50 species distributed in arid to sub-arid regions of the tropics and sub-tropics [2]. Among the cotton family, there are 5 traditional AD tetraploid species that are inter-crossable to various degrees, and 45 diploid species that are divided into 8 diploid genome groups (A, B, C, D, E, F, G, and K) [2]. There are four species have been domesticated for spinnable-fibre and collectively provide the world’s cotton production, including two diploids of *G. herbaceum* (A_1_; 2n = 2 × = 26) and *G. arboreum* (A_2_; 2n = 2 × = 26), which are Old World cottons from Asia-Africa, and two New World tetraploid cottons from the Americas, which are *G. hirsutum* or upland cotton (AD_1_; 2n = 4 × = 52) and *G. barbadense* or extra-long staple fibre cotton (AD_2_; 2n = 4 × = 52) [2]. Tetraploid cottons are presumably derived from a single polyploidization event that occurred 1–2 million years ago (MYA) between ancestors of *G. arboreum* (A_2_) and *G. raimondii* (D_5_) [3], accounting for 98% of the world’s cotton production.

Upland cotton is the primary source for cotton production, representing over 95% of the cotton fibre produced in the world [4]; however, its genetic diversity is narrow. *G. barbadense* has been used to improve fibre quality in upland cotton, as the second domesticated tetraploid cotton. However, it has some limitations, such as low yield and limited adaption. On the contrary, wild cotton germplasms harbour extensive genetic diversity and potential practicability, and they have rich sources of novel traits and are currently being mined to improve many beneficial agronomic traits. For example, *G. tomentosum* contains many unique agronomic traits, including insect-pest resistance, salt tolerance, heat tolerance, drought tolerance, nectarilessness and lint colour. *G. mustelinum* has fine fibre quality and Verticillium wilt resistantce. *G. darwinii* has many excellent traits, such as drought tolerance, finer fibre fineness, Fusarium wilt and Verticillium wilt resistance [5]. In addition, with the rapid development of next-generation sequencing (NGS) technologies, the recent publications on the genome sequence of the tetraploid AD_1_-genome [6, 7] and AD_2_-genome [8] and the diploid A-genome [9] and D-genome [10, 11] have improved the development of new analyses and comparative approaches for the genomics of both diploid and polyploid cottons.

Single nucleotide polymorphisms (SNPs) are known to be of considerable importance because they have a much higher abundance in the genome and are used to determine the population structure and for linkage disequilibrium (LD) analysis [12]. Similarly, insertions/deletions (InDels) have been used in rice, cotton and chickpeas for fine mapping and marker-assisted selection [13–15]. Furthermore, gene expression and function could be affected by the position of SNPs and InDels within a genome [12]. For example, variations present in coding regions and regulatory sequences may change protein functions and induce/repress gene expression. Therefore, the discovery of polymorphisms is very important in the study of genomic variation in crop species. To date, SNP discoveries have been applied in other crops beyond cotton [16]. SNP discoveries in cotton have progressed using different methods, such as BAC-end sequences [16], single copy sequences [17], transcriptome sequencing [18], reduced representation libraries (RRL) techniques [19]. Nevertheless, based on these techniques, access to information on SNPs is still very limited. In addition, the resources of wild cotton have not yet been excavated due to the large and complex genome, which greatly hinders the genetic research and cotton breeding.

Fortunately, with the rapid developments and applications of NGS technologies, many complexity reduction approaches have been developed based on NGS platforms, such as genotype-by-sequencing (GBS), 2b-RAD and RRLs. Therefore, with the successful application of these methods, a large number of sequence polymorphisms have been detected, including SNPs and InDels [20]. Recently, based on double barcode genotyping systems and deep sequencing, SLAF-seq was developed, which is an accurate and cost-effective high-throughput sequence-based technology [21]. More importantly, the SLAF-seq does not depend on the sequence of the reference genome, and it reduces the complexity of the reference genome, which is particularly important for species with an unknown genome [21].

Here, the SLAF-seq method was used to identify DNA polymorphisms between the reference (TM-1) [6] and 5 tetraploid cotton species, i.e., *G. hirsutum* cv. Emian22 (AD_1_), *G. barbadense* acc. 3–79 (AD_2_), *G. tomentosum* (AD_3_), *G. mustelinum* (AD_4_) and *G. darwinii* (AD_5_) genomes. Using the SLAF-seq data, an integrated analysis was carried out. Therefore, there were three main objectives in this study. First, interspecific variations were explored and characterized. Second, the phylogenetic trees of tetraploid cotton species were reconstructed. Third, the interspecific SNP and InDel markers were developed for future introgression breeding. In summary, the new DNA polymorphisms presented in this study will dramatically increase the efficiency for future research into high-density interspecific mapping, introgression breeding, genetic dissection and gene utilization in cotton.

## Results

### Build variation resources of tetraploid cottons

In the present study, a new variation resource was generated that had direct relevance to the current scenario of cotton breeding strategies. Using an Illumina high-throughput sequencing platform, a total of 117,795,823 80-bp long paired-end reads were generated from the five tetraploid cotton species, including two cultivars and three wild species (Table 1). After quality filtering, 111,735,304 high-quality reads were obtained, which varied from 18.6 to 26.3 million for the different materials. Nearly 80% of the high-quality reads were mapped to the cotton reference genome, which covered approximately 6.4% of the total genome for each material (Table 1). We also developed 467,735 SLAF tags among the five cotton species. SLAF tags were compared, polymorphic SLAF tags were identified (Additional file 1: Table S1), and it was observed that the number of polymorphic SLAF tags were unevenly distributed between the A subgenome (At) and D subgenome (Dt). The average depth for *G. hirsutum* cv. Emian22 (Gh_E22), *G. barbadense* acc. 3–79 (Gb_3-79), *G. tomentosum* (Gt), *G. mustelinum* (Gm) and *G. darwinii* (Gd) were 28.80, 25.14, 21.12, 24.73, and 31.13, respectively.

**Table 1:** Summary of sequence data and mapping statistics on the TM-1 genome

Species	Gh_E22	Gb_3–79	Gt	Gm	Gd
Total reads	24,655,891	21,139,301	18,567,855	21,126,892	26,305,884
High-quality reads	24,650,571 (99.98%)	21,125,785 (99.98%)	18,559,296 (99.95%)	21,109,849 (99.92%)	26,289,803 (99.94%)
Sequencing depth (fold)	1.55	1.33	1.17	1.33	1.65
Total reads mapped	20,176,030 (81.85%)	16,491,915 (78.07%)	15,223,151 (82.02%)	17,143,486 (81.21%)	21,468,542 (81.66%)
Genome coverage (%)	5.97%	6.16%	7.13%	6.25%	6.14%
Reads mapped with MAPQ30	12,480,220 (61.86%)	9,104,756 (55.21%)	8,749,989 (57.48%)	9,833,467 (57.36%)	13,020,982 (60.65%)

*MAPQ*30 Mapping quality of 30

### Identification and characteristics of SNPs and InDels

**Fig. 1:**
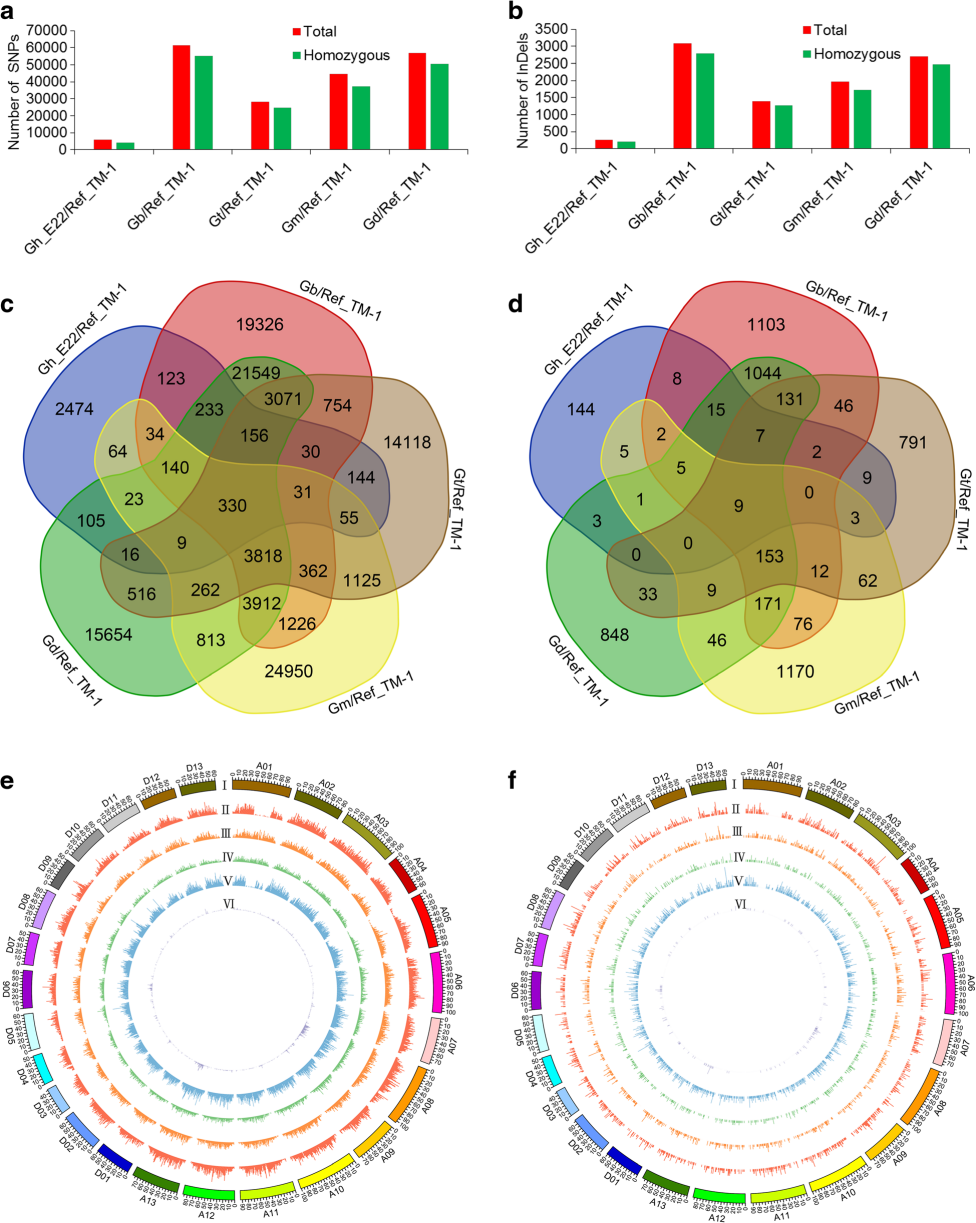
Distribution and number of SNPs and InDels detected in each of cotton species. **a**, **b** The number of SNPs (**a**) and InDels (**b**) detected in each of cotton species are illustrated in the bar graphs by different colours as indicated. Total number in each of cotton species is indicated by *red* bar graphs. The homozygous number in each cotton species is indicated by *green* bar graphs. **c**, **d** Overlap of SNPs (**c**) and InDels (**d**) across five species. **e**, **f** The distribution of SNPs (**e**) and InDels (**f**) detected in five cotton species (1 Mb window size). I: the chromosomes; II-VI: the total number of homozygous SNPs and InDels of Gd, Gm, Gt, Gb_3–79 and Gh_E22, respectively

Totally, 1,781,688 SNPs and 17,966 InDels were identified. Additionally, after filtering out the low confidence, a total of 139,176 polymorphisms, including 132,880 SNPs and 6,296 InDels, were detected between the reference TM-1 genome and the five tetraploid cotton species. Eventually, the total number of homozygous DNA polymorphisms was 120,692 for subsequent analysis, which included 114,833 SNPs and 5,859 InDels (Additional file 2: Figure S1). Further, the SNPs and InDels between each of the five tetraploid cotton species, and the reference TM-1 genome were also detected and showed that the Gb_3-79 had the highest DNA polymorphisms than other species, followed by Gd; the DNA polymorphisms of Gh_E22 were the least (Fig. 1a, b). Notably, the overlap of SNPs and InDels revealed a moderate degree of overlap among the five tetraploid cotton species (Fig. 1c, d).

**Fig. 2:**
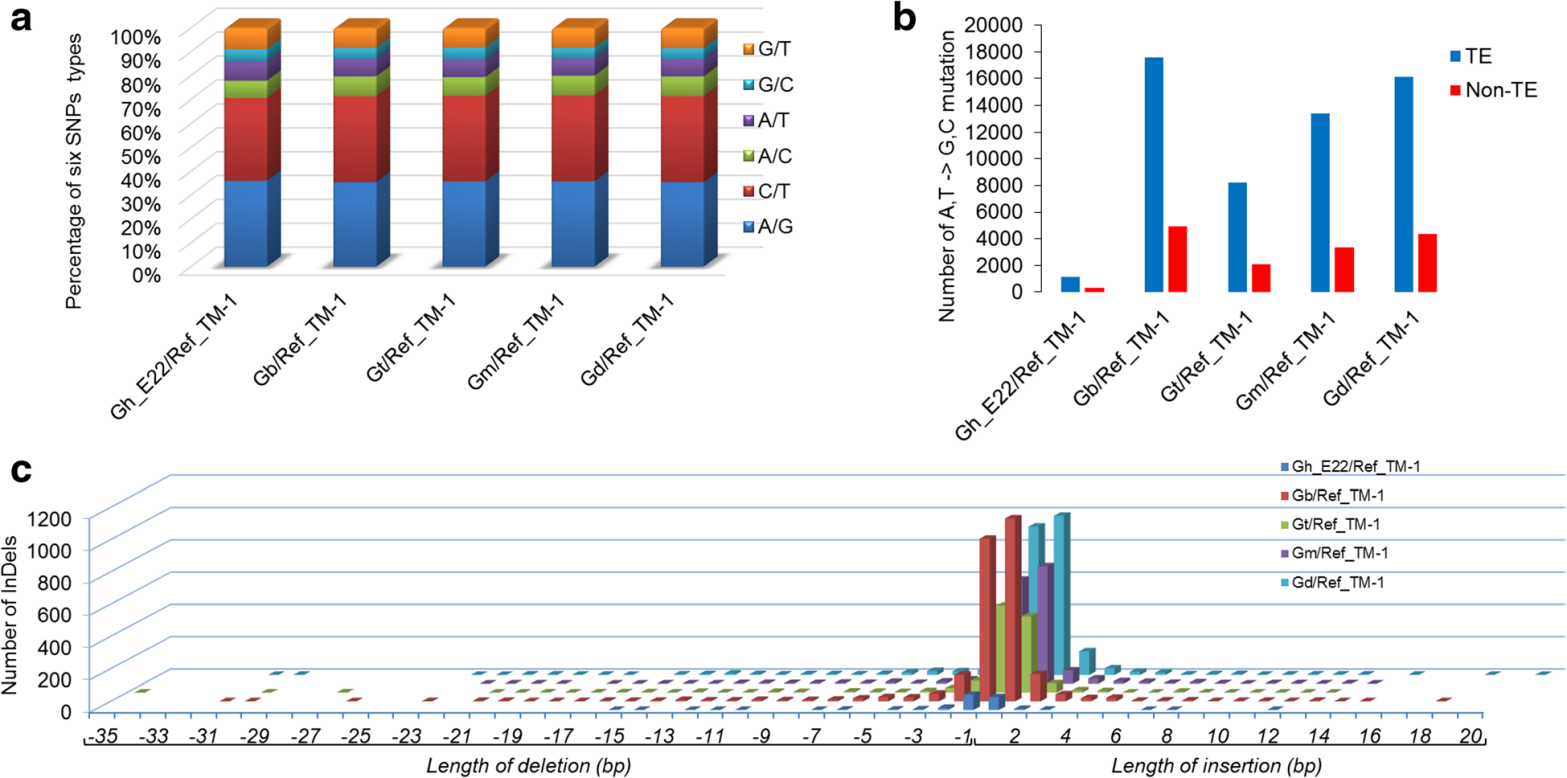
Percentage of SNPs substitution types, the number of AT-to-GC conversion in transposons, and length distribution of InDels among five cotton species. **a** Percentage of different SNPs substitution types in five cotton species. **b** The number of AT-to-GC conversion in transposons in five cotton species. TEs are indicated by *blue* bar graphs; Non-TEs are indicated by *red* bar graphs. **c** Length distribution of InDels among five cotton species. The number of insertions and deletions were shown on the y-axis in the bar graph. The numbers of various lengths (bp) are shown on the x-axis in the bar graph. Gh_E22/Ref_TM-1, Gb/Ref_TM-1, Gt/Ref_TM-1, Gm/Ref_TM-1 and Gd/Ref_TM-1, respectively indicates DNA polymorphisms identified between Gh_E22, Gb_3-79, Gt, Gm, Gd and the reference genome *G. hirsutum* acc. TM-1

The SNPs and InDels that were detected in each of the five cotton species were further analysed. The SNPs were classified as transitions (A/G and C/T; Ts) and transversions (A/C, A/T, G/C and G/T; Tv) based on nucleotide substitutions (Fig. 2a). Among the transitions, the proportions of A/G were slightly higher than C/T transitions in Gh_E22, Gt, and Gm, while the proportions of A/G was slightly lower than C/T transitions in Gb_3-79 and Gd. For transversions, the percentage of T/A transversions was relatively higher than others, namely, A/C, G/T and C/G. The ratio between transitions and transversions (Ts/Tv) for Gh_E22, Gb_3-79, Gt, Gm and Gd were 1.42, 1.66, 1.91, 1.65 and 1.73, respectively. For InDels, the length of the insertions ranged from 1 bp to 20 bp; however, the length distribution of deletions was 1 bp to 35 bp (Fig. 2c). The majority of InDels were single nucleotides, accounting for 78.55%, di- to tetra-nucleotides were 15.51%, and the remaining 5.94% were ≥ 5 bp.

The DNA polymorphisms in transposable elements (TEs) were also explored in the five cotton species. The percentages of SNPs/InDels in TEs for Gh_E22, Gb_3-79, Gt, Gm and Gd were 76.89%/73.71%, 77.91%/68.97%, 79.77%/72.69%, 79.63%/72.27%, and 78.57%/72.89%, respectively. The number of SNPs and Indels was calculated in TE and non-TE regions (Additional file 3: Figure S2). Further, through the analysis of SNPs and InDels in TEs, it was found that the DNA polymorphisms mainly existed in long terminal repeat (LTR) retrotransposons, particularly gypsy-type (Additional file 4: Table S2). Additionally, AT-to-GC conversion was larger in the TE than non-TE regions, which was significantly outnumbered in Gb_3-79 than in other cotton species (Fig. 2b).

### Genomic distribution of SNPs and InDels across the reference TM-1 genome

To survey the landscape of DNA polymorphisms for each of the five cotton species, the distributions of DNA polymorphisms were obtained. The number of homozygous SNPs and InDels was detected for subsequent analysis, and their frequency (window size was 1 Mb) varied across all 26 chromosomes. Herein, SNPs were observed to be unevenly distributed among the chromosomes and between the At and Dt in each of cotton species, with more SNPs in the At than the Dt (Fig. 1e). Likewise, the distribution of InDels among the chromosomes and between the At and Dt was also not uniform (Fig. 1f).

In addition, the total number of DNA polymorphisms and their density (number per 1 Mb) varied across the 26 cotton chromosomes in the five cotton species, and the total number of DNA polymorphisms was found to be positively correlated with the chromosome length (Additional file 2: Figure S1; Additional file 5: Table S3). Among them, the maximum number of homozygous SNPs was found on chromosome A08 (7,715), and the minimum number was found on chromosome D09 (2,278; Additional file 2: Figure S1b). However, for InDels, the maximum number was 341 on chromosome A06, and the minimum number was 130 on chromosome D01 (Additional file 2: Figure S1c). The distribution of gene densities across chromosomes from the TM-1 reference genome was also calculated, which showed the lowest gene density was in the middle of the chromosome (Additional file 2: Figure S1a). In addition, the genomic distribution of DNA polymorphisms was determined by calculating the frequency of occurrence (Additional file 2: Figure S1a). The highest frequency of homozygous SNPs was on chromosome A08 (74.5/Mb), and the lowest frequency of SNPs was on chromosome D09 (44.7/Mb; Additional file 5: Table S3). Similarly, the homozygous InDel frequency was the highest on chromosome A06 (3.31/Mb), and the lowest on chromosome D01 (2.12/Mb; Additional file 5: Table S3). Similarly, the total numbers of SNPs and InDels were unevenly distributed amomg the chromosomes and between At and Dt, with fewer DNA polymorphisms in the D-subgenome than the A-subgenome (Additional file 2: Figure S1b, c). Furthermore, a total of 607 pairs of homeologous genes containing SNPs were identified between At and Dt in the reference TM-1 genome, including 255 pairs of homeologous genes with one containing SNPs in At, and the other gene did not contain SNPs in Dt; 313 pairs of homeologous genes with one containing SNPs in Dt, and the other gene did not contain SNPs in At; and 39 pairs of homeologous genes with genes containing SNPs in both At and Dt. (Additional file 2: Figure S1a).

### Characterization and functional significance of SNPs and InDels

**Fig. 3:**
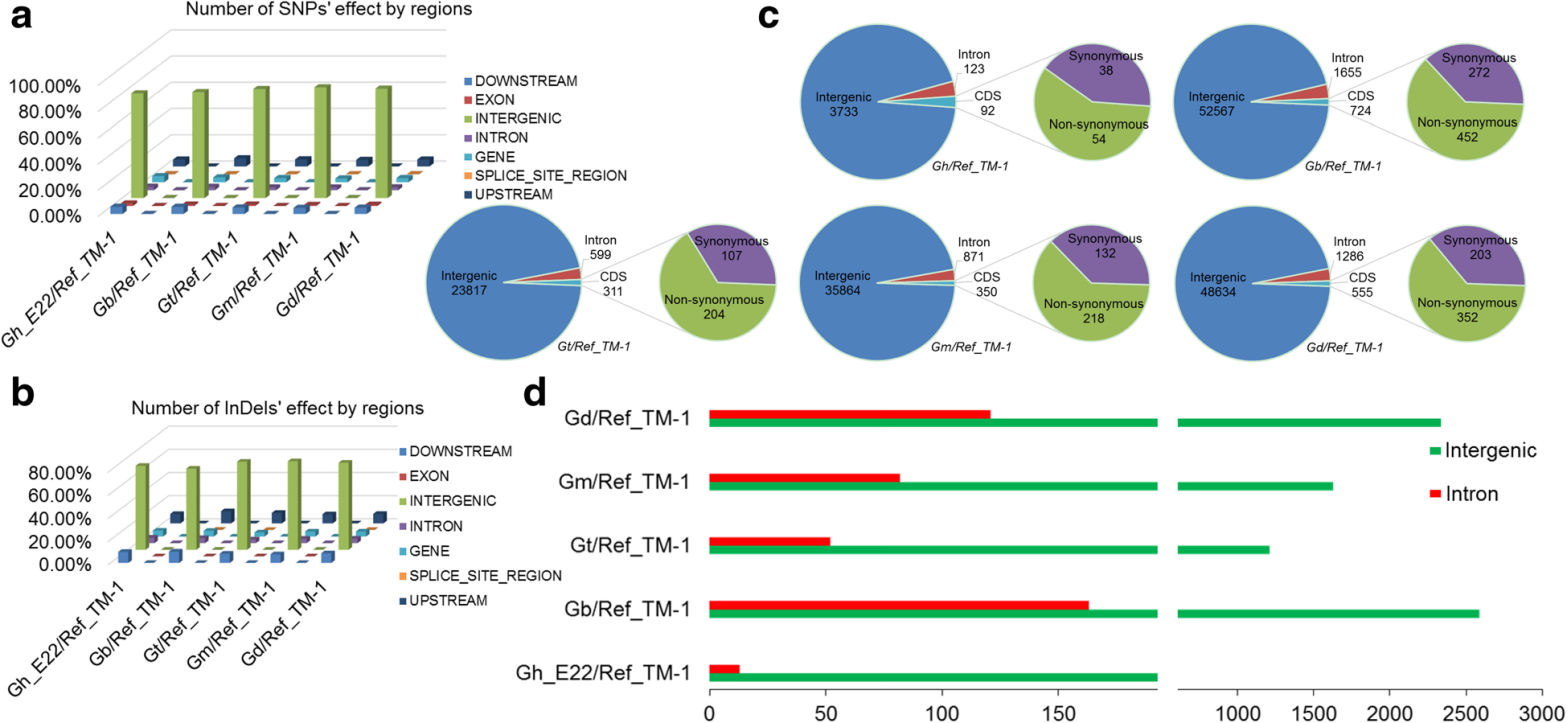
Annotation of SNPs and InDels. **a**, **b** Distribution of SNPs (**a**) and InDels (**b**) in different genomic regions for the five cotton species. **c**, **d** Distribution of SNPs (**c**) and InDels (**d**) in different intergenic and genic regions among the five cotton species. In CDS region, the number of synonymous and non-synonymous SNPs detected has also been shown among the five cotton species

Using the annotation of the cotton reference genome (TM-1), the distributions of SNPs and InDels within various genomic features were revealed. In general, a similar distribution pattern between SNPs and InDels was observed in the five species (Fig. 3a, b). The percentage of SNPs in genic regions ranged from 2.92 to 4.69%, while the percentage of InDels ranged from 4.37 to 5.15%, among the five cotton species. However, a substantial portion of SNPs (79.87 to 84.52%) and InDels (70.06 to 75.11%) were identified in the intergenic regions. A significant percentage of SNPs (9.85 to 11.73%) and InDels (15.36 to 11.73%) were also detected in the 5 kb upstream (promoter) and 5 kb downstream regulatory regions among the five cotton species (Fig. 3a, b).

**Fig. 4:**
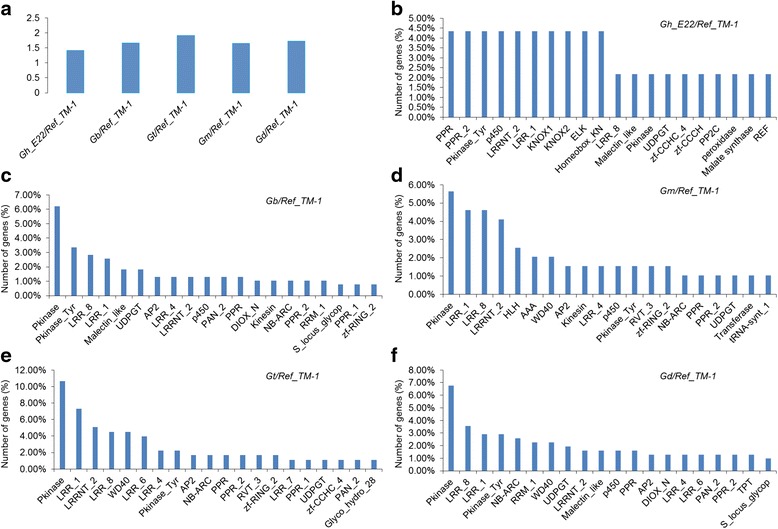
The ratio of non-synonymous to synonymous SNPs, and frequency of top 20 PFAM domains represented in the genes containing non-synonymous SNP among the five cotton species. **a** The ratio of non-synonymous to synonymous SNPs among five cotton species. **b**-**f** Frequency of top 20 PFAM domains represents in the genes containing non-synonymous SNP in Gh_E22, Gb_3-79, Gt, Gm and Gd, respectively

To further investigate the effect of SNPs in the coding sequence (CDS) region (Fig. 3c), amino acid substitution was analysed and a large proportion was detected to be non-synonymous among the five cotton species. The ratio of non-synonymous to synonymous SNPs was approximately 1.42 for Gh_E22, 1.66 for Gb_3-79, 1.91 for Gt, 1.65 for Gm, and 1.73 for Gd (Fig. 4a). Further, these non-synonymous SNPs were present in 46, 387, 178, 195, and 310 genes for Gh_E22, Gb_3-79, Gt, Gm, and Gd, respectively (Additional file 6: Table S4). The non-synonymous SNPs resided in the genes containing leucine-rich repeats (LRRs), pentatricopeptide repeats (PPRs), protein kinases, protein tyrosine kinases, WD domain repeats and NB-ARC domains (Fig. 4b-f). Among the five cotton species, 46 large-effect SNPs and 43 large-effect InDels were found in a total of 89 genes (Additional file 7: Table S5), which also contained many important functional domains, such as zinc-finger and P450 domains.

**Fig. 5:**
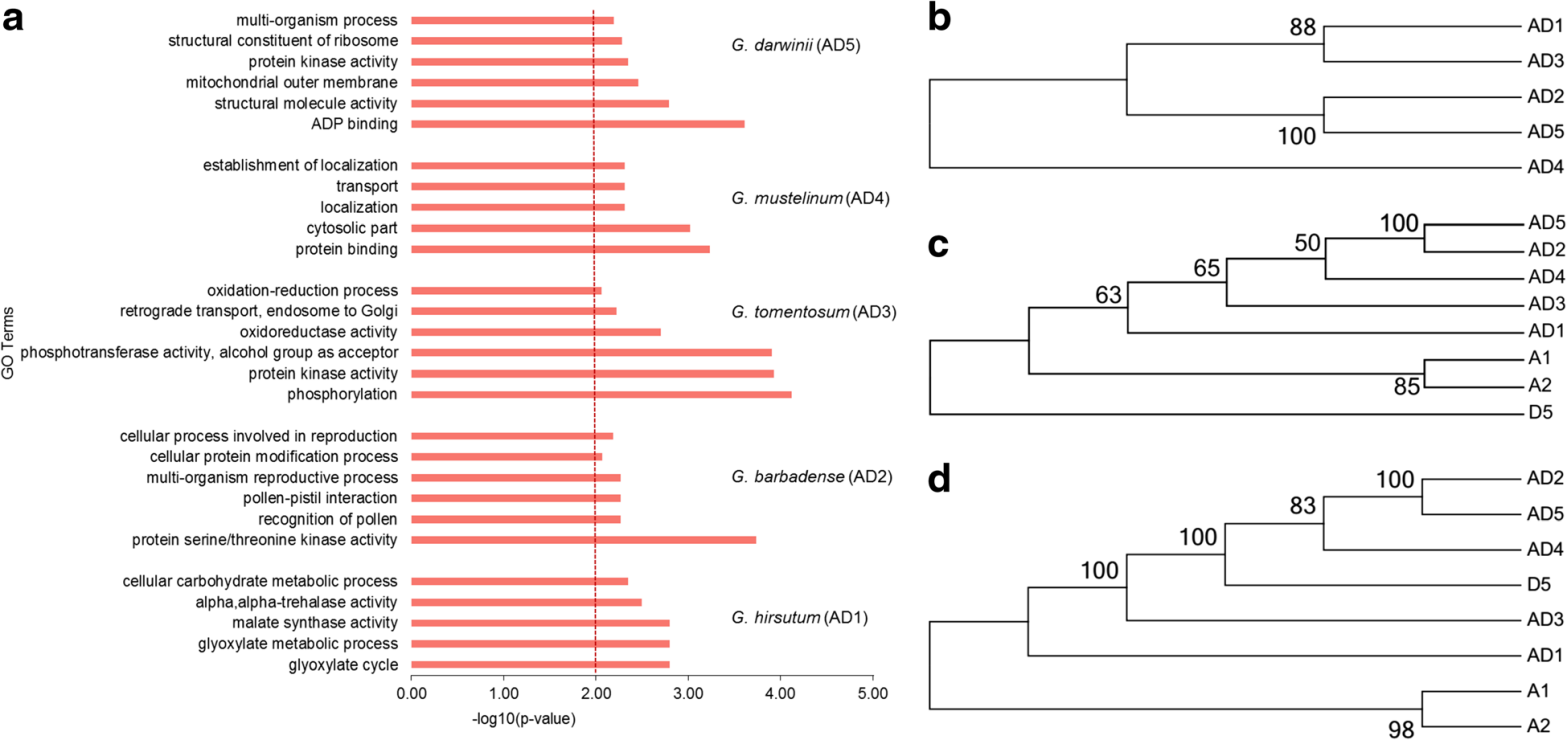
Significantly enriched GO terms and phylogenetic tree among five cotton species. **a** The significantly enriched GO terms are identified among five cotton species. **b** Phylogenetic tree was constructed among five cotton species. **c** Phylogenetic tree of A-homoeologs was constructed among five cotton species. **d** Phylogenetic tree of D-homoeologs was constructed among five cotton species

Gene ontology (GO) enrichment analysis revealed putative functions of the genes containing non-synonymous SNP variations among the five cotton species. Based on the reference genome annotation, a number of significantly enriched GO terms in each of the three main categories (biological processes, molecular function and cellular components) were identified. The significantly enriched GO terms, which were genes involved in biological processes, molecular function and cellular component of the GO classification, revealed striking differences between the five cotton species (Additional file 8: Table S6). For example, in Gb_3-79, the significant terms were related to reproduction, including multi-organism reproductive processes (GO:0044703), recognition of pollen (GO:0048544), pollen-pistil interaction (GO:0009875), pollination (GO:0009856), and reproductive processes (GO:0022414; Fig. 5a). In other cotton species, they also had their own unique characteristics; for instance, oxidoreductase activity (GO:0016491) and oxidation-reduction process (GO:0055114) were significantly enriched in Gt, which was associated with stress resistance (Fig. 5a). In Gm, many significantly enriched categories were mainly associated with tRNA, which was involved in the synthesis of protein (Additional file 8: Table S6). Significant GO terms of Gd were highly concentrated in cellular components, including the organelle outer membrane (GO:0031968), ribonucleoprotein complex (GO:0030529) and intracellular parts (GO:0044424; Additional file 8: Table S6).

### Phylogenetic analysis of the cotton diploids and polyploids

Understanding the phylogenetic relationships among the various genomes is not only significant for genetics research and breeding but also essential for conducting comprehensive studies of the plant genomes, especially in genome organization, function and evolution. To further study the phylogenetic relationships in different cotton genomes, phylogenetic trees of the cotton diploids and polyploids were constructed. Specifically, and unsurprisingly, the five tetraploid cotton species were classified into three branches, one consisting of Gh_E22 (AD_1_) and Gt (AD_3_), one consisting of Gm (AD_4_), and the third containing Gb_3-79 (AD_2_) and Gd (AD_5_; Fig. 5b). Additionally, two sets of SNPs were generated, and the phylogenetic trees for the At and Dt homoeologs were constructed, independently (Fig. 5c, d). For the A-subgenome, D_5_ clade as one outgroup species, it showed a high concordance in the phylogeny of the cotton diploids and polyploids. The concordance analysis identified three main branches, which were the A-subgenomes of five tetraploid cotton clades, [A_1_ + A_2_] clade and D_5_ clade (Fig. 5c). Compared with A-homoeologs, the [A_1_ + A_2_] clade was one outgroup species, and analysis results of D-homoeologs showed that the clades were more consistent with the sister-species relationship. The D_5_ clade had closer relationships with the D-subgenomes of the five tetraploid cotton clades than [A_1_ + A_2_] (Fig. 5d).

### Interspecific variations for introgression genetics and breeding

To broaden the genetic basis of upland cotton, introgression line populations were developed in our laboratory, which used Gh_E22 as the receptor parent and Gb_3-79, Gm, Gt, and Gd as donor parents, respectively. The SNPs/InDels identified in this study facilitated the introgression genetics and breeding as molecular markers. Combined with our laboratory resources to investigate the extensive genetic diversity and the potential practicability, Gh_E22 vs. Gb_3-79, Gh_E22 vs. Gt, Gh_E22 vs. Gm and Gh_E22 vs. Gd were analysed.

**Fig. 6:**
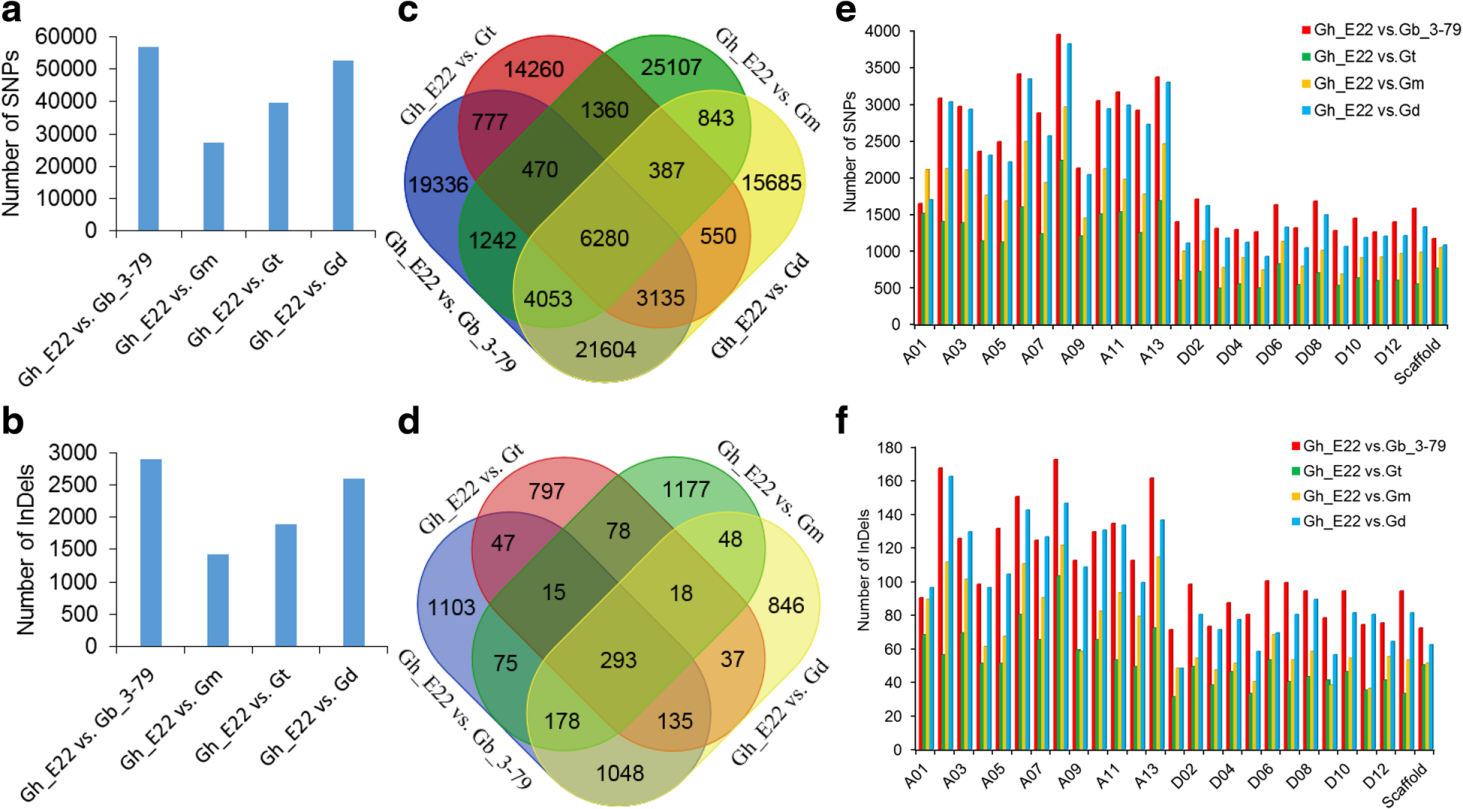
Distribution of SNPs and InDels between four Gh_E22 vs. Gb_3-79, Gh_E22 vs. Gt, Gh_E22 vs. Gm and Gh_E22 vs. Gd. **a**, **b** The total number of SNPs and InDels between different combinations are illustrated in the bar graphs. **c**, **d** The overlap of SNPs and InDels. **e**, **f** Distribution of SNPs and InDels on each chromosome are illustrated in the bar graphs in different combinations

To gain insight into the differences in various combinations, the total number of homozygous SNPs/InDels was identified between Gh_E22 vs. Gb_3-79, Gh_E22 vs. Gt, Gh_E22 vs. Gm and Gh_E22 vs. Gd, which were 56,897/2,894, 27,219/1,420, 39,742/1,882 and 52,537/2,603, respectively (Fig. 6a, b; Additional file 9: Table S7), observing that Gh_E22 vs. Gb_3–79 had the higher DNA polymorphisms than the others. Further, overlaps of the SNPs/InDels among the four combinations were shown with a Venn diagram (Fig. 6c, d). The overlapped SNPs/InDels between Gh_E22 vs. Gb_3–79 and Gh_E22 vs. Gd were larger than other two combinations. The unique DNA polymorphisms were maximum in Gh_E22 vs. Gm (26,284), and minimum in Gh_E22 vs. Gt (15,017). Intriguingly, the SNPs/InDels were not evenly distributed cross the chromosomes. The SNPs/InDels in the At were more frequent than those in the Dt (Fig. 6e, f). The Gh_E22 vs. Gb_3–79 had the highest DNA polymorphisms on each chromosome, followed in descending order by Gh_E22 vs. Gd, Gh_E22 vs. Gm and Gh_E22 vs. Gt, except for the chromosome A01, where Gh_E22 vs. Gd had the maximum SNPs (96) and Gh_E22 vs. Gt had the maximum InDels (68; Fig. 6e, f). The SNPs/InDels on A08 were the maximum in the four combinations, which were 3,939/172, 2,225/103, 2,955/121, 3,810/146 in Gh_E22 vs. Gb_3–79, Gh_E22 vs. Gt, Gh_E22 vs. Gm and Gh_E22 vs. Gd, respectively. In contrast, the minimum number of SNPs were on D11 (1,250) in Gh_E22 vs. Gb_3–79, D03 (489) in Gh_E22 vs. Gt, D09 (679) in Gh_E22 vs. Gm and D05 (921) in Gh_E22 vs. Gd. However, the minimum number of InDels, were on D01 (71) in Gh_E22 vs. Gb_3–79, D01 (31) in Gh_E22 vs. Gt, D11 (36) in Gh_E22 vs. Gm and D01 (48) in Gh_E22 vs. Gd, respectively (Fig. 6e, f).

The randomly selected 40 SNPs on chromosome A01 were used to design primers to check the gel-based polymorphism. Based on the SSCP analysis, there were 5 polymorphic markers (50%) between Gh_E22 and Gb_3–79, 1 (10%) between Gh_E22 and Gt, 2 (20%) between Gh_E22 and Gm, and 3 (30%) between Gh_E22 and Gd.

## Discussion

In the present study, a high quality of 111,735,304 80-bp long paired-end reads and 467,735 SLAFs were generated using high-throughput SLAF-seq. The average sequencing depth of each cotton species was 26.18×. Additionally, a total of 114,833 homozygous SNPs, and 5,859 homozygous InDels were identified. Interspecific DNA polymorphisms for Gb_3–79, Gt, Gm and Gd occurred at a much higher rate compared to intraspecific DNA polymorphisms for Gh_E22 (Fig. 1a, b), especially in Gb_3–79, which may be that they belong to different cotton species and the distant phylogenetic relationships. Gb_3–79 and Gd were found to have more overlap than the other three species, and a similar phenomenon also occurred in the following two species, Gh_E22 and Gt, which may be related to their genetic relationship (Fig. 1c, d). As is known, the conventional five polyploid cotton species have been divided into three branches by previous cotton researchers; one branch is *G. hirsutum* and *G. tomentosum*, the other is *G. mustelinum*, and the third is *G. barbadense* and *G. darwinii* [2]. Our analysis, as a new resource, provides further evidence on the accuracy of the previous studies in their phylogenetic relationships.

For the SNPs, the non-synonymous/synonymous SNP ratios in different cotton species were 1.42–1.91 (Fig .4a), which was slight higher than IR64/Pokkali (1.19) and IR64/N22 (1.15) in rice [12], and JS-335 (1.11) and UPSM-534 (1.10) in soybean [22]. In addition, the frequency of transitions was found to be significantly higher than transversions. Transition bias has been reported in rice, maize, chickpeas, and loquat [12, 23–25]. The proportions of C/T were slightly lower than A/G transitions in Gh_E22, Gt and Gm, while the proportions of C/T were slightly higher than A/G transitions in Gb_3–79 and Gd, which may be associated with divergence times. Similar observations were previously reported in *G. hirsutum* and *G. barbadense* [20]. However, the percentage of T/A transversions was relatively higher than others, namely, A/C, G/T and C/G. Similar observations were detected in rice and citrus [23, 26], which is remained to be explained as an unexpected observation. The ratio of transitions and transversions (Ts/Tv) for Gh_E22, Gb_3–79, Gt, Gm and Gd were 2.315, 2.487, 2.498, 2.516 and 2.483, respectively, which were slightly higher than *G. hirsutum* (2.194) and *G. barbadense* (2.210) of a previous report in cotton [16], IR64/Pokkali (2.340) and IR64/N22 (2.370) in rice [12], and JS-335 (1.978) and UPSM-534 (1.961) in soybean [22]. Further, AT-to-GC conversion in Gb_3–79 significantly outnumbered Gh_E22, followed in descending order by Gd, Gm and Gt (Fig. 2b), which could provide the potential DNA methylation sites, if occurring in TEs [27].

However, as for InDels, the length distribution of InDels was found to be larger than that in rice [12] and less than that in soybean [22]. Intriguingly, InDels in each of the individual five cottons also showed an upward bias towards single nucleotide insertions and deletions, which was similar to rice and soybean [12, 22]. The abundance of SNPs and InDels were detected in 5 kb upstream and 5 kb downstream regulatory regions [8], which may be due to the non-coding regulatory regions having lower sequence conservation and levels of purifying selection pressure than coding regions [12]. In animals, the variations in the cis-regulatory regions have demonstrated the importance in the regulation of gene expression [28–30]. In plants, a few studies also have shown that such genetic variations play a major role in the regulation of gene expression and agronomic traits [31–33].

Functional annotation of these genes containing variations revealed putative characteristics in different cotton species (Fig. 5a). In Gb_3–79, a number of the significant terms were related to reproduction (Fig. 5a), which suggested that the variations may result in the disruption of regulatory genes determining reproductive fertility [34, 35] and may help to explain the hybrid breakdown and low yield in offspring between *G. barbadense* and *G. hirsutum*.

In previous studies, the genus *Gossypium* has been researched broadly in phylogeny and a consensus phylogenetic tree was generated [2, 36]. The phylogenetic analysis has been reported in a previous study [37], and it was based on the targeted genes sequence capture method. Here, we used high-throughput sequencing to reconstruct the phylogenetic trees that showed a high concordance with the historically hypothesized phylogeny, providing strong support for the phylogenetic relationships in diploid and allotetraploid cottons (Fig. 5b-d).

Interspecific introgression breeding is a traditional method and requires labour, time and funds; however, it can be expedited by molecular marker-assisted selection. In a recent study, the cotton breeding potential of introgression lines between *G. hirsutum* and *G. barbadense* was investigated for its yield and fibre quality improvement, including heterosis, combining ability and genetic effects [38]. Similarly, the introgression line populations were constructed in our laboratory with Gh_E22 as the receptor and the other tetraploid species as donors. Currently, only 5,152 gel-based loci were identified between Gh_E22 and Gb_3–79 [39]. As is known, the gel-based markers are time-consuming and have low efficiency. In this study, a huge number of SNPs and InDels identified, plus the high throughput platform, will facilitate and speed up the genetics and breeding of the introgression lines (Additional file 9: Table S7). Although few polymorphisms were detected from the randomly selected 40 SNP markers on A01, because the SSCP is still a gel-based genotyping method and has low resolution, we do believe that more polymorphisms will be detected with a high-resolution detecting platform, such as the SNP chip or genotyping by resequencing.

## Conclusions

A large number of high-quality DNA polymorphisms through SLAF-seq were identified in five tetraploid cotton species. Further comprehensive analysis of DNA polymorphisms provided valuable insights into the different tetraploid cotton species, especially between cultivated and wild cottons. To better utilize the novel characteristics of drought tolerance, defence responses and salt stress tolerance, etc. in wild cottons, numerous interspecific variations were identified here, which will be very practical in future introgression breeding. Overall, the comprehensive data generated in this study provided insights into cotton evolution and a resource for future researches in high-density interspecific mapping, introgression breeding improvement in cotton.

## Methods

### Plant materials and DNA extraction

Five tetraploid cotton species were employed in our study: *G. hirsutum* cv. Emian22 (2n = 4 × = 52, AD_1_), *G. barbadense* acc. 3–79 (2n = 4 × = 52, AD_2_), *G. tomentosum* (2n = 4 × = 52, AD_3_), *G. mustelinum* (2n = 4 × = 52, AD_4_), and *G. darwinii* (2n = 4 × = 52, AD_5_), which were denoted hereafter as Gh_E22, Gb_3-79, Gt, Gm and Gd, respectively. To investigate the evolutionary relationship between diploid and polyploid cottons, three additional diploid genomic sequence reads were also analysed, which were *G. herbaceum* (2n = 2 × = 26, A_1_), *G. arboreum* (2n = 2 × = 26, A_2_), and *G. raimondii* (2n = 2 × = 26, D_5_) [10, 40].

The elite cultivar Gh_E22 and the genetic and cytogenetic standard line Gb_3–79 [41] were grown in 2014 at the experimental field of Huazhong Agricultural University, Wuhan, China. The frozen leaves of the other three cotton species were collected from the National Wild Cotton Nursery (Sanya, China). Fresh young leaves from each of five cotton species were frozen in liquid nitrogen immediately, and stored in a − 70 °C freezer. The genomic DNA of the five species was isolated from the fresh leaves of a single plant per species using the Plant Genomic DNA Kit (TIANGEN Biotech, Beijing, China). DNA concentrations were estimated with a NanoDrop 2000C Spectrophotometer (Thermo Scientific, USA), and the quality was subsequently evaluated by electrophoresis on a 1% agarose gel.

### SLAF sample preparation for high-throughput sequencing

The SLAF libraries were constructed based on the result of a pre-designed scheme, which was used to determine the optimized restriction enzymes and sizes of restriction fragments to optimize SLAF yields and obtain the maximize SLAF-seq efficiency. Three criteria were considered in the pre-design experiment: (i) The final number and length of the SLAFs must be suitable for the specific experimental system and must meet the expected one; (ii) The distribution of SLAFs must be even in the genome; and (iii) The repeated SLAFs must be avoided. The procedure followed was as described previously by Sun et al. [21], with minor modifications. Firstly, an appropriate restriction enzyme combination, *RsaI* + *HaeIII* (NEB, Ipswich, MA, USA) were selected to digest purified genomic DNA. To maintain sequence depth uniformity, the objective fragments of 314 ~ 344 bp in size were selected. Secondly, an adenine nucleotide (A) overhang was added to the digested fragments. The dual-index paired-end adapters’ ligation, the adapter-modified ends obtainment and polymerase chain reaction (PCR) were carried out step by step. Subsequently, PCR products were purified using an E.Z.N.A.® Cycle Pure Kit (Omega, London, UK) and the purified PCR products were incubated at 37 °C with MseI, T4 DNA ligase, ATP, and Solexa adapter. The reaction products were purified with a Gel Extraction Kit (Qiagen, Hilden, Germany) and electrophoresed on a 2% (w/v) agarose gel [21]. Thirdly, pared-end reads were generated for analysis by high-throughput sequencing in an Illumina HiSeq™ 2500 system (Illumina, Inc., San Diego, CA, USA) according to the manufacturer’s recommendations at Biomarker Technologies Corporation in Beijing [21].

### Read mapping and discovery of SNPs and InDels

To assess the initial quality of the raw sequence data, the software of FastQC was used (http://www.bioinformatics.babraham.ac.uk/projects/fastqc/). All reads were processed for more stringent quality control and filtered. Those reads containing adaptor/primer contamination and low-quality bases were removed. The sequence of the reference genome TM-1 was downloaded from the CottonGen database (https://www.cottongen.org). Subsequently, the high-quality trimmed paired-reads were mapped onto the cotton reference genome TM-1 using Burrows-Wheeler Aligner (BWA) software (v0.7.10) [42]. The mapping output of BWA was processed by sorting and duplicated marking using Samtools [43] and Picard (http://broadinstitute.github.io/picard/). Only the mapped reads with high mapping quality (MQ ≥ 20) and high base quality (Q ≥ 30) were considered for downstream analysis.

DNA polymorphisms were called with the Genome Analysis Toolkit (GATK, v3.1.1) software [44], Samtools/bcftools [43, 45], respectively. Further, the common SNPs and InDels were obtained using default parameters. The stringent parameters of the software were used to minimize detection of the false positives when calling SNPs and InDels. SNPs and InDels were filtered with the criteria that the minimum read depth was less than 10, and the average base quality was less than 30. The functions of Realigner Target Creator and InDels-Realinger in GATK (v3.1.1) [44] were used to realign InDels, and Unified Genotyper was used to identify genotypes across the five species using the parameters of “-stand_call_conf 30.0”. To further remove low confidence SNPs and InDels, the following stringent parameters were applied: (−−clusterSize 3 --clusterWindowSize 10 --filter “DP < 10” --filterName LowDPFilter --Filter “ MQ0 > = 4 && ((MQ0/(1.0 * DP)) > 0.1)” --filterName LowMQFilter).

### DNA polymorphisms analysis

The genomic distribution of DNA polymorphisms was analysed and visualized with Circos software [46], and the density of DNA polymorphisms in each 1 Mb interval on the 26 cotton chromosomes was calculated. To assess the distribution in different genomic regions, their positions were integrated with a GFF3 file containing cotton genome annotation. For analysis of the genomic distribution and annotation of SNPs and InDels, in-house perl scripts were carried out. The overlap of DNA polymorphisms identified in multiple species was shown with a Venn diagram (http://bioinformatics.psb.ugent.be/webtools/Venn/). The synonymous and non-synonymous SNPs, and large-effect SNPs and InDels were identified with *G. hirsutum* (TM-1) [6] as a reference using the single-nucleotide polymorphism effect predictor software [47]. To identify homologous genes containing SNPs, BLASTX with a cutoff e-value of 10^−10^ and MCscan software [48] with default settings were used.

### Construction of phylogenetic trees

It is known that allopolyploid cottons contain two homologous subgenomes, the A-genome and D-genome donors. According to previous reports, the At donor was likely the two extant A genome species, *G. herbaceum* (A_1_) and *G. arboreum* (A_2_); while the closest extant diploid of the Dt donor was similar to *G. raimondii* (D_5_) [49]. At and Dt were separately used as references, and the genome sequences of the extant diploid species of *G. herbaceum* (A_1_), *G. arboreum* (A_2_) and *G. raimondii* (D_5_) [10, 40] were separately mapped. Next, the common positions of SNPs between diploid species and the At and Dt were obtained using customized perl scripts. Finally, two sets of SNPs for the A- and D- homoeologs were generated independently.

The software SNPhylo [50] and MEGA6.0 [51] were used to construct phylogenetic trees of the diploid and polyploidy cotton species. Using SNPphylo [50] software, reliable and accurate phylogenetic trees were easily constructed and analysed. Then, the software MEGA6.0 [51] was used to draw the phylogenetic tree image.

### Functional categorization and comparative analysis

To explore variations, we focused on analysing the putative functions of the genes. Based on the cotton genome annotation, the putative functions of the genes were assigned. The conserved domain (s) was predicted by searching the cotton genome annotation. The Gene Ontology enrichment analysis was performed using Fisher’s exact test in Blast2GO version 2.8 [52] with a *p*-value cut-off of ≤0.01.

### Interspecific marker development and validation

Interspecific markers between Gh_E22 and the other four species were identified. To assess and validate the SNPs for future applications, a total of 40 SNPs on chromosome A01 for Gh_E22 vs. Gb_3-79, Gh_E22 vs. Gt, Gh_E22 vs. Gm and Gh_E22 vs. Gd were randomly selected to design flanking primers. These flanking primers were designed from the 150 bp flanking sequences of the 40 randomly selected SNPs using BatchPrimer3 [53] with an optimal annealing temperature of 57 °C, optimal product size of 200 bp and the remaining parameters at their default settings. The designed primers were synthesized by Beijing Tianyi Huiyuan Life Science and Technology, Inc. (Wuhan, China). PCR amplifications and silver staining were carried out as previously described by Lin et al. [54]. The PCR products were separated on 8% native polyacrylamide gels using SSCP technology [55].

## Additional files

Additional file 1: Table S1. Distribution statistics of SLAFs and polymorphic SLAFs on each chromosome. (XLS 25 kb)

Additional file 2: Figure S1. Distribution and total number of SNPs and InDels detected on the cotton chromosomes. (a) The distribution of SNPs and InDels detected on all the 26 chromosomes of cotton (1 Mb window size). I: the chromosomes; II: gene density; III: the total number of homozygous SNPs; IV: the total number of homozygous InDels; V: the homologous genes between At and Dt indicated by different colours including SNPs. SNPs in At is indicated by red line, Dt indicated by yellow line, SNPs in both At and Dt indicated by blue line. (b, c) The number of SNPs (b) and InDels (c) detected on each cotton chromosome are illustrated in the bar graphs by different colours. Total number on each cotton chromosome is indicated by red bar graphs. The homozygous number on each cotton chromosome is indicated by green bar graphs. (TIF 5665 kb)

Additional file 3: Figure S2. The number of SNPs and InDels in TE and non-TE regions in different cotton species. (a) The number of SNPs in TE and non-TE regions. (b) The number of InDels in TE and non-TE regions. (TIF 551 kb)

Additional file 4: Table S2. Statistics of SNPs and InDels detected in transposable elements among the five cotton species. (XLS 24 kb)

Additional file 5: Table S3. Frequency of SNPs and InDels detected among the five cotton species on individual cotton chromosome. (XLS 28 kb)

Additional file 6: Table S4. Non-synonymous SNPs present in the coding region of genes among the five cotton species. (XLS 220 kb)

Additional file 7: Table S5. List of large-effect SNPs and InDels and genes harboring at least one large-effect SNPs and InDels in the five cotton species (provided as separate file). (XLS 40 kb)

Additional file 8: Table S6. The significantly enriched GO terms in each cotton species. (XLS 40 kb)

Additional file 9: Table S7. List of the DNA polymorphisms in Gh_E22 vs. Gb_3-79, Gh_E22 vs. Gt, Gh_E22 vs. Gm and Gh_E22 vs. Gd. (XLS 11624 kb)

## Abbreviations

At: A subgenome
BWA: Burrows-wheeler aligner
CDS: Coding sequence
Dt: D subgenome
GATK: Genome analysis toolkit
GBS: Genotype-by-sequencing (GBS)
GO: Gene ontology
InDels: Insertions/Deletions
LD: Linkage disequilibrium
LRR: Leucine-rich repeat
LTR: Long terminal repeat
Mb: Megabasse
MYA: Million years ago
NB-ARC: Nucleotide-binding adaptor shared by APAF-1, R proteins, and CED-4
NGS: Next-generation sequencing
PPR: Pentatricopeptide repeat
RRLs: Reduced representation libraries (RRLs)
SLAF-seq: Specific length amplified fragment sequencing
SNPs: Single nucleotide polymorphisms
SSCP: Single-strand conformation polymorphism
TEs: Transposable elements
Ts/Tv: Transitions/Transversions
