# Supplementary figures and images for: Uncovering SNP and indel variations of tetraploid cottons by SLAF-seq
Source: BMC Genomics. 2017 Mar 23;18:247. doi: 10.1186/s12864-017-3643-4 (PMC5363057; doi:10.1186/s12864-017-3643-4)

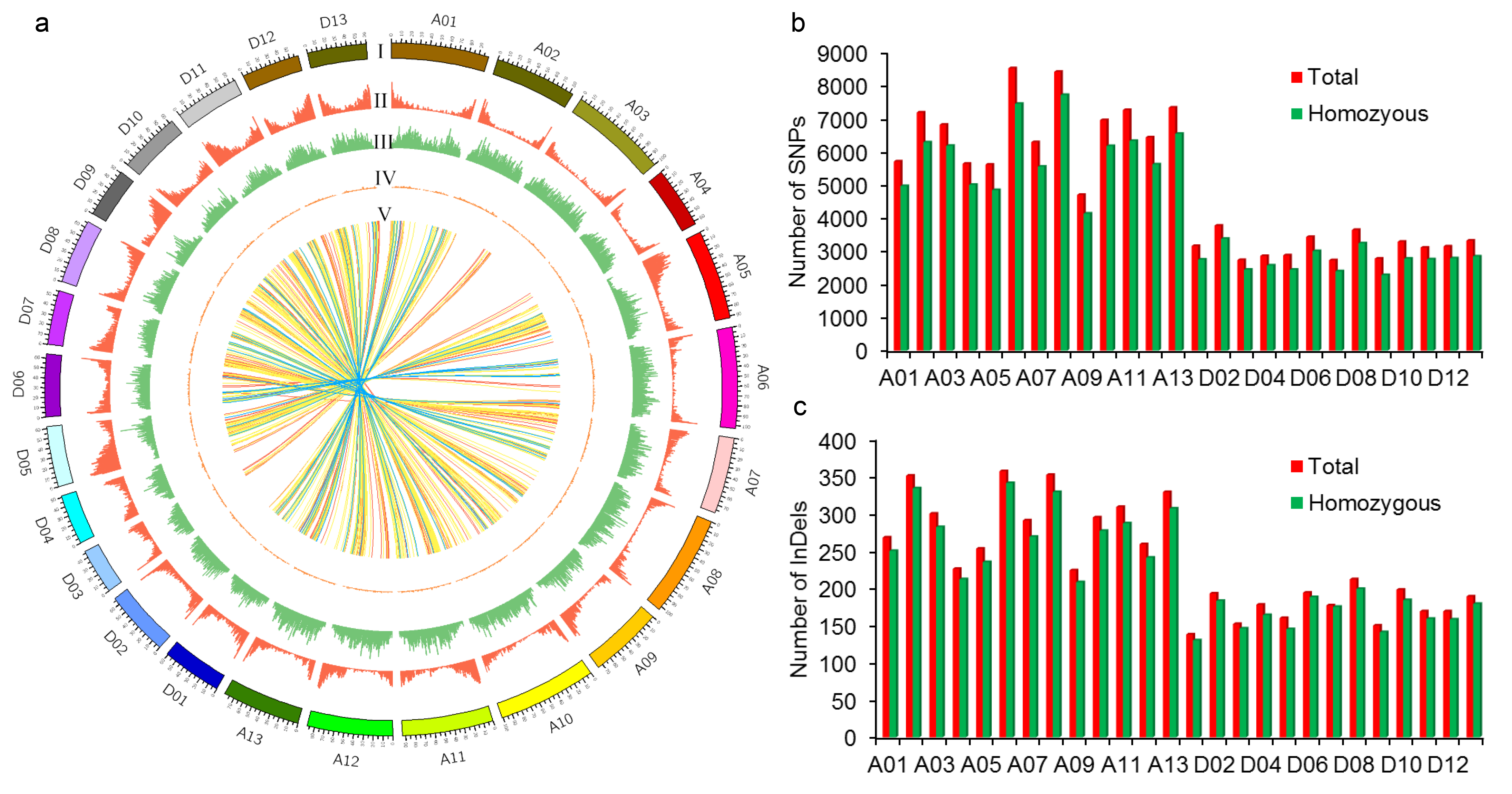

Supplement: Supplementary file 2 — Distribution and total number of SNPs and InDels detected on the cotton chromosomes. (a) The distribution of SNPs and InDels detected on all the 26 chromosomes of cotton (1 Mb window size). I: the chromosomes; II: gene density; III: the total number of homozygous SNPs; IV: the total number of homozygous InDels; V: the homologous genes between At and Dt indicated by different colours including SNPs. SNPs in At is indicated by red line, Dt indicated by yellow line, SNPs in both At and Dt indicated by blue line. (b, c) The number of SNPs (b) and InDels (c) detected on each cotton chromosome are illustrated in the bar graphs by different colours. Total number on each cotton chromosome is indicated by red bar graphs. The homozygous number on each cotton chromosome is indicated by green bar graphs. (TIF 5665 kb) [file 12864_2017_3643_MOESM2_ESM.tif]

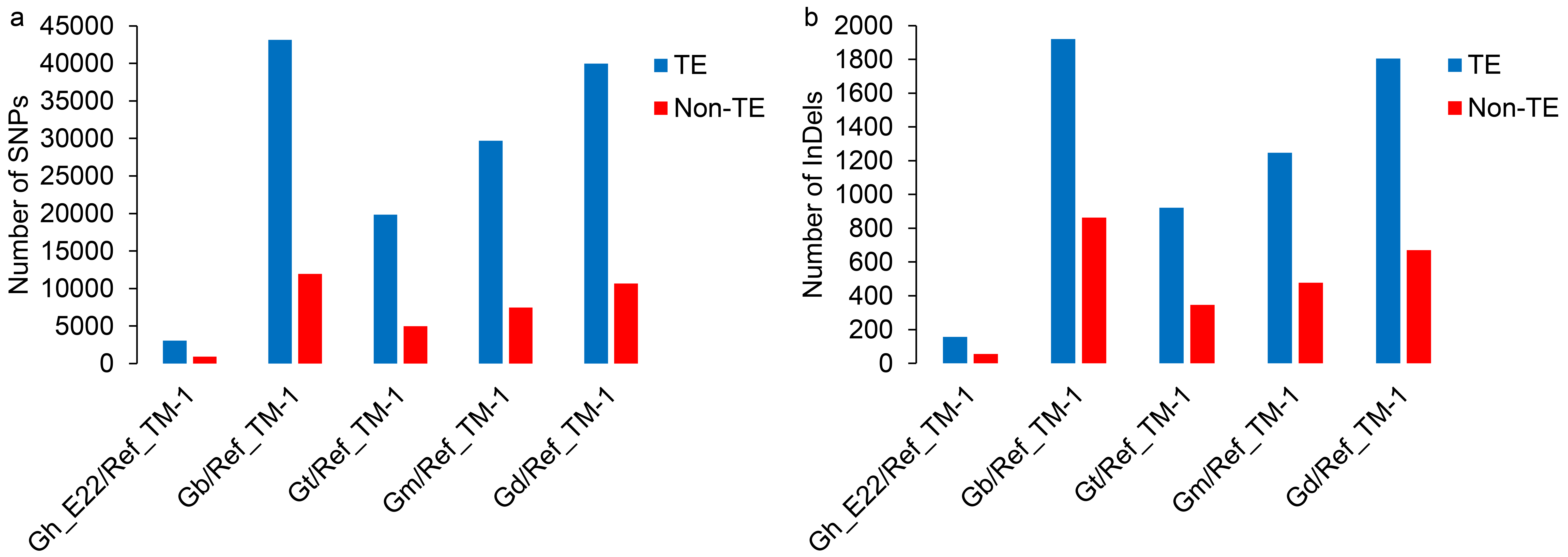

Supplement: Supplementary file 3 — The number of SNPs and InDels in TE and non-TE regions in different cotton species. (a) The number of SNPs in TE and non-TE regions. (b) The number of InDels in TE and non-TE regions. (TIF 551 kb) [file 12864_2017_3643_MOESM3_ESM.tif]
